# Seasonality of coronavirus shedding in tropical bats

**DOI:** 10.1098/rsos.211600

**Published:** 2022-02-09

**Authors:** Léa Joffrin, Axel O. G. Hoarau, Erwan Lagadec, Olalla Torrontegi, Marie Köster, Gildas Le Minter, Muriel Dietrich, Patrick Mavingui, Camille Lebarbenchon

**Affiliations:** Université de La Réunion, UMR Processus Infectieux en Milieu Insulaire Tropical (PIMIT), INSERM 1187, CNRS 9192, IRD 249, GIP CYROI, 2 rue Maxime Rivière, Saint Denis, La Réunion, France

**Keywords:** alpha-coronavirus, disease ecology, longitudinal study, Indian Ocean, Molossidae, Reunion Island

## Abstract

Anticipating cross-species transmission of zoonotic diseases requires an understanding of pathogen infection dynamics within natural reservoir hosts. Although bats might be a source of coronaviruses (CoVs) for humans, the drivers of infection dynamics in bat populations have received limited attention. We conducted a fine-scale 2-year longitudinal study of CoV infection dynamics in the largest colony of Reunion free-tailed bats (*Mormopterus francoismoutoui*), a tropical insectivorous species. Real-time PCR screening of 1080 fresh individual faeces samples collected during the two consecutive years revealed an extreme variation of the detection rate of bats shedding viruses over the birthing season (from 0% to 80%). Shedding pulses were repeatedly observed and occurred both during late pregnancy and within two months after parturition. An additional shedding pulse at the end of the second year suggests some inter-annual variations. We also detected viral RNA in bat guano up to three months after bats had left the cave. Our results highlight the importance of fine-scale longitudinal studies to capture the rapid change of bat CoV infection over months, and that CoV shedding pulses in bats may increase spillover risk.

## 1. Introduction

Host–pathogen interactions are dynamic and may depend on host life cycle (reproduction, parturition, dispersal of young and migration) and on abiotic factors such as climate or seasonal variations (precipitation, droughts, temperatures and cyclones) [1–5]. Analysing the effect of such factors on animal infections will contribute to deciphering transmission cycles of infectious agents in natural ecosystems. For example, a 6-year survey of a wild blue tit population showed that infection with *Plasmodium relictum* mainly occurs during postnatal dispersal events [6]. Longitudinal studies of wild animal populations are thus important in disease ecology, with applications to assess risks of spillover of zoonotic infectious agents [5,7].

Seasonal viral shedding or infection has been reported in bats for many pathogens belonging to different viral families (e.g. Paramyxoviridae, Filoviridae, Coronaviridae and Astroviridae) [7–12]. Such seasonality in viral infection dynamics in bats is often driven by host population structure and their reproductive cycles. These cycles can be highly synchronized and may create conducive conditions for viral transmission between hosts (i.e. aggregation of pregnant females and parturition increasing the size of the colony) and infection within hosts (e.g. immune function during gestation and lactation, waning of maternal antibodies in juveniles) [13–16].

Longitudinal studies have been conducted to assess shedding dynamics of viruses associated with bats [7,11,17–19]. For instance, a significant variation in the prevalence of Marburg virus infections in Egyptian fruit bats (*Rousettus aegyptiacus*) has been reported throughout the breeding and the birthing seasons [7]. An increase in Marburg virus prevalence after parturition suggests a major role of juvenile bats in the transmission. More recently, it has been shown that immunity and colony size/density-dependent transmission lead to multi-year fluctuations in Nipah virus transmission intensity among *Pteropus* bats in Bangladesh [20].

Although bat coronaviruses (CoVs) have been detected worldwide [21], the ecological factors involved in CoV infection dynamics remain to be precisely assessed. It has been suggested that parturition and age could be important drivers in bat CoV transmission dynamics [17,18,22,23]. Such studies are currently needed given that bat-associated CoVs can be the progenitors of viruses responsible for outbreaks in humans and livestock (e.g. severe acute respiratory syndrome CoV-2, Middle East respiratory syndrome CoV, swine acute diarrhoea syndrome virus). Only a few bat species have been investigated for CoV shedding dynamics at fine scale [17,18], and further investigations are thus needed, especially in tropical areas.

Reunion free-tailed bats (RFTB; *Mormopterus francoismoutoui*) have recently been identified as hosts for an *Alphacoronavirus* (alpha-CoV) [24]. This small tropical insectivorous species is endemic to Reunion Island [25] and roosts in large monospecific colonies in natural caves and buildings (i.e. bridges and houses [24,25]), especially during the birthing season (austral summer) when females seasonally aggregate in maternity colonies to give birth [11]. Several thousands of individuals, with up to 1000 individuals per square metre, can be found in RFTB colonies [11,26].

We conducted a 2-year longitudinal study of the largest RFTB maternity colony to investigate the effect of the bat colony structure (reproductive status and age) on CoV detection in faeces. We based our sampling scheme on a fine temporal scale (intervals of 2–3 weeks) to capture rapid changes in CoV shedding in faeces. We monitored the colony during the whole period of occupation (nine months each year) and tested (i) whether changes in the colony structure (reproductive status and age) during the birthing season could affect the prevalence of bats shedding CoV, in particular with the addition of newborns and juvenile bats, (ii) if similar patterns of infection occur between years and (iii) if the level of accumulation and persistence of CoV RNA in the environment (dry guano) was associated with temporal variation in CoV shedding in fresh faeces.

## 2. Material and methods

### 2.1. Study site

The study was conducted from 2016 to 2018, in a natural cave (30 m^3^) on the west coast of Reunion Island, occupied by bats from October to June. The bat colony is monospecific and mostly composed of adult females during the early stages of the birthing season (October–December). Previous studies have estimated between 40 000 and 50 000 flying adult bats to be present in the cave before parturition. After the parturition period (starting in mid-December), the population includes lactating females, neonates and juveniles. As the adults progressively leave the cave (late January–March), the remaining population comprises mostly juveniles, which subsequently leave by May, resulting in the cave being empty from June to September [11,27]. In this study, we assessed population age structure and parturition timing based on the presence of adults (brown fur), newborns (nude pink skin) and juveniles (dark grey fur) [27].

### 2.2. Collection of biological material

A non-invasive sampling strategy was set up to avoid direct manipulation of bats and to limit colony disturbance (electronic supplementary material, figure S1) [27]. Samples were collected every two to three weeks between June 2016 and September 2018. Fresh faeces were collected inside the cave in the morning, by placing benchguard^®^ sheets (60 cm × 49 cm) underneath the roosting bats. To avoid sampling bias associated with potential spatial variation in bat CoVs shedding inside the colony, seven different locations within the cave were selected at the beginning of the study and were coded as ‘sheet no. 1’ to ‘sheet no. 7’. During each sampling session, benchguard^®^ sheets were placed in these locations if bats were present, resulting in sample collection from one to five of the seven defined areas for each sampling event. The objective was to avoid sampling bias associated with potential spatial variation in bat CoVs shedding inside the colony. During each sampling session, between 12 and 50 fresh droppings were collected on each benchguard^®^ sheet within 15 min after fresh faeces were dropped and placed in individual virus transport medium (VTM) [28]. Samples were maintained refrigerated in the field and stored at −80°C.

In parallel, during each sampling session, we also collected dry guano samples from the narrowest part of the cave entrance, at 10 different locations with 50 cm separation from each other, along with a 4.5 m transect (electronic supplementary material, figure S2). Each location was coded with a letter from A to J, leading to the collection of 10 samples for each sampling session (electronic supplementary material, figure S3). Sterile open-ended 2 ml syringes were used to collect a core sample to obtain *ca* 130 mg of guano. Guano sample was immediately mixed with 1.5 ml of VTM and kept refrigerated in the field and stored at −80°C. Details regarding dry guano sampling design are available in Joffrin *et al*. [27].

### 2.3. Coronavirus detection in faeces and guano

Samples were briefly vortexed and then centrifuged at 1500*g* for 15 min. RNA was extracted from 140 µl of each sample's supernatant using a QIAamp Viral RNA mini kit (QIAGEN, Valencia, CA, USA) and eluted in 60 µl of AVE buffer. Reverse transcription was performed with 10 µl of RNA using ProtoScript II Reverse Transcriptase and Random Primer 6 (New England BioLabs, Ipswich, MA, USA) as previously described [28]. cDNAs were tested for the presence of the RNA-dependent RNA-polymerase (RdRp) gene using a multi-probe real-time (RT) PCR targeting a 179 bp length fragment [29]. RT-PCR was performed with ABsolute Blue QPCR Mix (Thermo Fisher Scientific, Waltham, MA, USA) and 2.5 µl of cDNA in a CFX96 Touch™ Real-Time PCR Detection System (Bio-Rad, Hercules, CA, USA) [24].

For each maternity season, a subset of CoV positive faeces (2016–2017, 30%; 2017–2018, 20%) was sequenced by direct Sanger method on both strands by Genoscreen (Lille, France). Sequences were edited with Chromas Lite v.2.6.4 software [30] and aligned with CLC Sequence Viewer 8.0 software [31], with the same reference sequences used in a previous study [24].

### 2.4. Statistical analysis

We explored the effect of sampling collection date (years, months and weeks), sampling collection sheet (no. 1 to no. 7) inside the cave for fresh faeces, sampling location along the transect (A–J) for dry guano on the probability of CoV detection. We used generalized additive mixed models (GAMMs) with binomial error distributions using the *mgcv* package in R v. 3.5.1 software [32] to model binomial prevalence data as a smoothed function of time (months and weeks) while accounting for sampling location (collection sheet inside the cave and sampling location along the transect) as explanatory variables. The model with the best Akaike information criterion (AIC) score was selected.

We also explored the effect of CoV shedding in fresh faeces on the probability of CoV detection in the guano from October 2016 to June 2018. After checking the autocorrelation of the variables by a Durbin–Watson test (*car* package) and the normality of the residuals by a Shapiro–Wilk test for each maternity season, we used the Spearman test and calculated coefficient rho (*ρ*) to determine the correlation between the CoV shedding in faeces and the number of positive guano samples at the cave entrance for each sampling season. Rho values were interpreted as follows: 0.00–0.39 ‘weak’ correlation, 0.40–0.59 ‘moderate’ correlation, 0.60–0.79 ‘strong’ correlation and 0.80–1.00 ‘very strong’ correlation. Coefficients were considered significant for *p*-value < 0.05. Analyses were conducted with R v. 3.5.1 software [32].

## 3. Results

### 3.1. Reunion free-tailed bats population dynamic and structure

**Figure 1.**
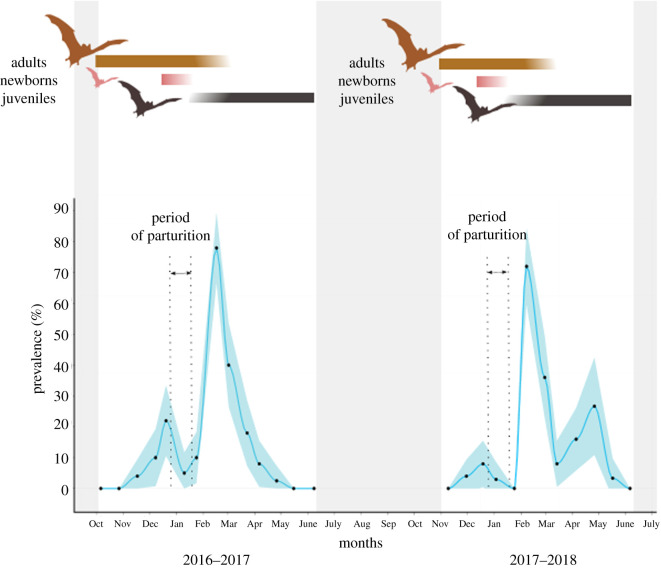
Prevalence of Reunion free-tailed bats ( *Mormopterus francoismoutoui*) shedding CoV in faeces during two consecutive maternity seasons (October 2016–June 2018). The blue shaded area represents the 95% confidence interval. Dots indicate sample collection dates. Grey areas correspond to periods without bats in the cave. The presence of adults, newborns and juveniles in the colony is indicated at the top of the figure: brown bats corresponds to adult, pink bats corresponds to newborns, and black bats to juveniles.

During our visual monitoring of the colony, the first adult bats were spotted inside the cave in October 2016 and in November 2017 (figure 1). More than half of the cave surface was covered with bats three to six weeks after the arrival of the first colonizing bats and it lasted for two to three months (November–February). Then, occupancy progressively dropped until all bats had left the cave in June. Newborns were sighted from late December onwards, for about a month for the two sampling campaigns. By the end of January, the colony was mainly composed of juvenile bats with no newborns and only a few adults sighted. These observations were consistent with the previous study on this colony [11,26]

### 3.2. Coronavirus shedding in fresh faeces

Overall, 1080 individual faeces were collected during 26 sampling sessions for two consecutive years. A total of 179 faeces tested positive for CoV (16.6%). None of the faeces tested positive for CoV at the beginning and at the end of both maternity seasons (i.e. during the first and the last sampling sessions).

We observed a first increase in bats shedding CoV at the beginning of the birthing season, with a prevalence (±95% confidence interval) of 22.0 ± 11.5% in 2016–2017 and 8.0 ± 7.5% in 2017–2018 (figure 1). This prevalence then dropped to 5.0 ± 6.8% in 2016–2017 and to 0% in 2017–2018. A second major increase in the prevalence of bats shedding CoV was observed several weeks after the first one, reaching 78.0 ± 11.5% in 2016–2017 and 72.0 ± 12.4% in 2017–2018. In 2016–2017, the prevalence decreased from March onward, until all bats left the cave. In 2017–2018, however, a third increase was detected at the end of the season (26.7 ± 15.8%). Based on AIC score, the best-supported GAMM included the sampling week and the sampling sheet as significant variables (electronic supplementary material, table S3). Fresh faeces were significantly more likely to be CoV positive during weeks 6, 7 and 9 (i.e. in February) as compared to other weeks (respectively, *p* < 0.001, *p* < 0.001 and *p* < 0.05; electronic supplementary material, figure S4), supporting a seasonal increase in bat shedding CoV at this time of the year. Increases of bats shedding CoV during other periods of the year were not significant despite the important variation in the prevalence of bats shedding CoV in December and in March (figure 1). However, this result is likely to be biased by our sampling scheme: samples were not collected in the same weeks during the two monitored seasons. An effect of sampling sheet was also detected for sheet no. 6 (*p* < 0.01), very likely reflecting a bias in sampling effort. Indeed, the location of sheet no. 6 was only sampled for two sampling sessions during weeks 4 and 7, leading to an overestimation of CoV prevalence for this location.

No genetic variation was observed on the 45 partial RdRp sequence fragments (179 bp). Sequences were 100% similar to the alpha-CoV previously detected in RFTB on Reunion Island (sequence accession number MN183188).

### 3.3. Coronavirus RNA in dry guano

**Figure 2.**
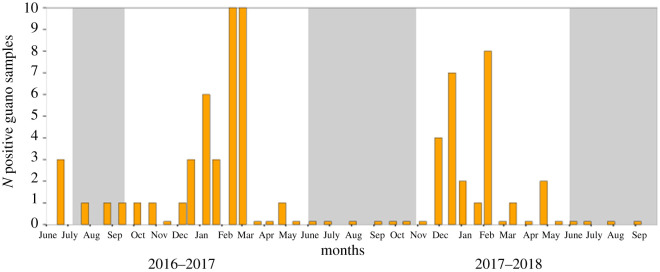
Number of positive guano samples at each sampling session. Grey areas correspond to time of the year without bats in the colony.

Overall, 380 guano samples were collected at the cave entrance during two consecutive years (190 each year), from June 2016 to September 2018 (10 samples collected along a transect in 38 individual sampling sessions; electronic supplementary material, table S2). In total, 67 guano samples (17.6%) tested positive for the presence of CoV (figure 2). Dry guano samples were found CoV positive all during the year, even when the cave was unoccupied. Interestingly, guano samples collected in July, August and September 2016 tested positive for CoV (electronic supplementary material, table S2) although bats had left the cave by June at the latest. Based on AIC scoring, the best-supported GAMM reveals a significant effect of time and location on the probability of CoV detection in dry guano, with sampling months and weeks as a significant factor (*p* < 0.005; electronic supplementary material, table S4). Overall, a significant decrease of the detection of positive samples was observed after week 9 (*p* < 0.001; electronic supplementary material, table S4). An effect of sampling location on CoV detection was found, with a slightly higher number of positive samples detected from location H than other locations (*p* < 0.05; electronic supplementary material, table S4 and figure S5).

We found a moderate positive correlation between the probability of CoV detection in fresh faeces and the number of positive dry guano samples at the cave entrance, for the first sampling season (*ρ* = 0.57, *p* < 0.05), but not for the second one (*ρ* = 0.35, *p* = 0.27).

## 4. Discussion

We investigated the infection dynamics of bat CoVs in a RFTB maternity colony during two consecutive years. Our results provide evidence that the prevalence of RFTB shedding alpha-CoV varies during the course of the birthing season, with a major shedding pulse occurring two months after parturition. Similar patterns were observed for both years, although an increase in the prevalence of RFTB shedding alpha-CoV was detected at the end of the second year. We did not find any spatial variation in bat shedding CoV prevalence inside the colony. Dry guano sampling revealed that viral RNA could be detected in the environment after all bats have left the cave; however, the infectivity of viral particles potentially maintained in bat guano remains to be assessed. Finally, the use of non-invasive sampling methods to explore the alpha-CoV infection dynamics in RFTB seems to be successful and efficient, adding another example to investigations conducted with limited impact on wild animals [11,17–19]. Although a recent study suggests that non-invasive sampling may lead to overestimation of the prevalence of bats shedding viruses [33], we nevertheless highlight that a detailed assessment of the temporal variation of CoV circulation in wild bats can be done with limited impact on the host population.

Major variations in alpha-CoV shedding occurred during the birthing season. Such infection dynamics has previously been observed for other alpha-CoVs in *Myotis myotis* bats in the temperate region [17], and also for other infectious agents in RFTB [11]. Dietrich *et al*. investigated *Leptospira* spp. bacteria and paramyxovirus infection dynamics in RFTB urine over the birthing season in the same colony [11]. The prevalence of these two pathogens increased at the beginning of the birthing season (mid-December), and three months after the parturition period (late March) [11]. Together, these results suggested that the first increase in prevalence could result from a transient pregnancy-related depressed immunity [11]. However, the major shedding pulse we detected for alpha-CoV does not seem to coincide with the second peak observed for *Leptospira* bacteria and paramyxovirus. These distinct patterns may reflect differences in population immunity towards these infectious agents, or differences in the transmission rates between the different pathogens. Seasonal breeding and birthing in high-density colonies lead to a substantial increase in the number of susceptible individuals, therefore enhancing viral transmission [1,5,34]. Moreover, studies on virus transmission in bat colonies have suggested that newborns may be protected against infections by maternal antibodies [11,17,35,36]. The duration of antibody waning seems to vary between viruses; during henipavirus and filovirus infections, it has been estimated that maternal antibodies may persist for three to six months [13–15]. Even if no studies have investigated the duration of maternal antibodies of bat CoVs, one can hypothesize that the major epidemic wave we observed in juvenile bats may coincide with the waning of maternal antibodies [11,17]. Finally, differences in the sampling design (faeces versus urine, sampling inside the cave versus outside) and PCR sensitivity towards each of the infectious agents may also explain differences in infection dynamics between *Leptospira* bacteria, paramyxovirus and CoV in RFTB. Collecting material inside the cave also allows the sampling of all age groups (adults, newborns, non-flying juveniles and flying juveniles) over the maternity season, whereas sampling conducted outside the cave is restricted to flying bats. Further investigations would be required to precisely assess patterns of virus shedding at the individual level, based on bat capture rather than environmental sampling, and give a more accurate estimation of infection dynamics for the whole colony.

We identified an inter-annual variation of bats shedding CoV, with a shedding pulse at the end of the second year. At least two scenarios may explain this potential increase in the prevalence of shedding bats. First, it may reflect a desynchronization in parturition of pregnant females, leading to a second pool of susceptible individuals at the end of the maternity season. However, the absence of newborns in the colony after March 2018 does not support this hypothesis. Second, juvenile bats from other colonies may have prospected the cave as a new potential roost, leading to the introduction of new susceptible individuals to the colony and favouring virus transmission. Our study site is the most important known roosting site for RFTB which can host more than 40 000 individuals during the maternity season [11]. However, little is known about the geographic origin of the females aggregating at the beginning of the maternity season, nor the destination of mothers and juveniles after they leave the cave and more globally, how the RFTB population is structured at the island scale. Knowledge of the structure and connectivity of bat populations is important to understand infection dynamics and viral dispersion within and between colonies and may depend on ecological or physiological factors [37]. Thus, studies on population structure and population dynamics through population genetics (e.g. bat mitochondrial and nuclear DNA) and capture–mark–recapture may provide important information on RFTB seasonal movements and viral transmission between roosting sites.

No genetic variation was observed in the sequenced CoV RdRp, although we cannot exclude that this may be associated with the limited number of viruses we sequenced (25% of the PCR positive samples), the length of the amplicons (179 bp) and the conservative nature of the RdRp CoVs. Therefore, we cannot exclude the circulation of different CoV variants in the monitored RFTB colony, at different times in the season. Rare co-infections with multiple CoV strains have already been reported elsewhere, either with the same CoV group such as in Lyle's flying fox (*Pteropus lylei*) colony in Thailand and in Old World leaf-nosed bats (*Hipposideros* sp.) in Ghana, or with both alpha- and beta-CoVs in Egyptian fruit bats (*Rousettus aegyptiacus*) in Guinea [18,38,39]. To accurately explore the genetic diversity of CoVs and their evolution, the use of PCR systems targeting structural genes coding for the spike or envelope proteins is needed. Sequencing the aforementioned genes may provide useful information on phylodynamic studies of CoVs in their natural hosts, although it is challenging given the size and variability of the spike gene [40].

A higher proportion of CoV positive samples were found in fresh faeces than in dry guano, and the probability of detection of positive CoV guano samples did not reflect CoV dynamics in faeces. The use of environmental samples such as dry guano instead of fresh samples may then not be appropriate to study virus infection dynamics in bat colonies. Wacharapluesadee *et al*. [41] investigated CoV presence in dry bat guano in Thailand and only 3.8% of guano samples tested positive for CoV. As enveloped viruses are considered more sensitive to denaturation than non-enveloped viruses, full CoV viral particles may be rapidly degraded in the environment under the action of humidity, rain, temperature or ultraviolet exposure [42–46]. On the other hand, the detection of CoV RNA in bat guano before the birthing season, when the cave was empty, might suggest the environmental persistence of viral particles in guano over time. Previous experimental studies revealed that viruses such as other alpha-CoVs (i.e. transmissible gastroenteritis virus and murine hepatitis virus) can survive (i.e. replicate in host cell lines) up to three weeks at 25°C and several months at 4°C in water and pasteurized sewage, and even longer under low humidity on indoor surfaces [45,46]. These patterns also corresponded to those of *Betacoronavirus* (beta-CoVs) such as SARS-CoV-1 and SARS-CoV-2 [44,47–49]. Since most of these studies were focused on water detection and only a few on fomites, there is limited information available regarding CoV survival in soil. Environmental or indirect transmission of bat-borne pathogens to other hosts through the contamination of shared habitats has already been hypothesized for some viruses and bacteria [50]. In our study, the lack of virus isolation limits further interpretation about the potential of dry guano as a suitable route of transmission to other hosts. In addition, our estimation of CoV prevalence is based on RNA detection and may not reflect the prevalence of bats shedding infectious particles. Future experimental studies on CoV survival in dry bat guano under different abiotic conditions (temperature, humidity and ultraviolet radiation) may provide useful answers on viral infectivity duration in the environment.

Alpha- and beta-CoVs have also been reported in many other mammalian hosts [38–40]. Investigations on bat viruses and how they interact with their hosts in wild environments have considerably improved our knowledge of disease emergence but are still not sufficient for anticipating future outbreaks [51–53]. The development of ecological studies is still needed to determine the drivers and patterns in viral shedding and cross-species transmission [54] and to better assess the conditions, places and timing of emergence risk [55,56]. Environmental alterations such as biodiversity loss and land-use change are considered as key drivers of virus emergence [57,58]. Factors associated to these environmental changes (food scarcity, stress and pollution) may directly impact viral shedding dynamics at the individual and population levels [3,59,60]. Habitat fragmentation and human encroachment into wild areas enhance contact with infected wild animals, therefore facilitating spillover opportunities [57,58]. For a better anticipation of the emergence in other hosts, including humans, long-term eco-epidemiological studies are needed to precisely assess CoV transmission dynamics in wild reservoirs [57,61–63]. The recent SARS-CoV-2 emergence highlights the need for a better assessment of CoV diversity and spillover risk, at both local and regional scales.

## Data accessibility

The datasets supporting this article have been uploaded as part of the electronic supplementary material. The data are provided in the electronic supplementary material [64].
